# Assessing Free-Radical-Mediated DNA Damage during Cardiac Surgery: 8-Oxo-7,8-dihydro-2′-deoxyguanosine as a Putative Biomarker

**DOI:** 10.1155/2017/9715898

**Published:** 2017-06-04

**Authors:** Linda Turnu, Alessandro Di Minno, Benedetta Porro, Isabella Squellerio, Alice Bonomi, Chiara Maria Manega, José Pablo Werba, Alessandro Parolari, Elena Tremoli, Viviana Cavalca

**Affiliations:** ^1^Centro Cardiologico Monzino, IRCCS, Milan, Italy; ^2^Dipartimento di Chirurgia Cardiaca, IRCCS Policlinico San Donato, Milan, Italy

## Abstract

Coronary artery bypass grafting (CABG), one of the most common cardiac surgical procedures, is characterized by a burst of oxidative stress. 8-Oxo-7,8-dihydro-2′-deoxyguanosine (8-oxodG), produced following DNA repairing, is used as an indicator of oxidative DNA damage in humans. The effect of CABG on oxidative-induced DNA damage, evaluated through the measurement of urinary 8-oxodG by a developed and validated liquid chromatography-tandem mass spectrometry (LC-MS/MS) method in 52 coronary artery disease (CAD) patients, was assessed before (T0), five days (T1), and six months (T2) after CABG procedure. These results were compared with those obtained in 40 subjects with cardiovascular risk factors and without overt cardiovascular disease (CTR). Baseline (T0) 8-oxodG was higher in CAD than in CTR (*p* = 0.035). A significant burst was detected at T1 (*p* = 0.019), while at T2, 8-oxodG levels were significantly lower than those measured at T0 (*p* < 0.0001) and comparable to those found in CTR (*p* = 0.73). A similar trend was observed for urinary 8-iso-prostaglandin F_2*α*_ (8-isoPGF_2*α*_), a reliable marker of oxidative stress. In the whole population baseline, 8-oxodG significantly correlated with 8-isoPGF_2*α*_ levels (*r* = 0.323, *p* = 0.002). These data argue for CABG procedure in CAD patients as inducing a short-term increase in oxidative DNA damage, as revealed by 8-oxodG concentrations, and a long-term return of such metabolite toward physiological levels.

## 1. Introduction

Although coronary artery bypass grafting (CABG) is one of the most common cardiac surgical procedures, patients' morbidity and mortality, due to its adverse postoperative complications, are exceedingly high [1]. To investigate the mechanism underlying such adverse events, studies have assessed the biochemical effect of oxidative stress during this procedure [2, 3]. Reactive oxygen species (ROS), which are thought to be, at least in part, responsible for such adverse events, show a burst during CABG [4]. However, whilst the lipid peroxidation [5, 6] and the antioxidant defenses [7, 8] have been already evaluated, only limited information is available on oxidative DNA damage [9].

In human cells, the balance between DNA damage/repair is a finely regulated physiological process, resulting in approximately 10^4^ oxidized DNA bases eliminated for cell for day in healthy subjects [10]. The oxidative modification of DNA by ROS can generate a variety of possible DNA lesions. Among nucleobases, guanine has the lowest redox potential, thus, being the most susceptible to oxidation [11]. Indeed, its stable urinary end-product, 8-oxo-7,8-dihydro-2′-deoxyguanosine (8-oxodG), is one of the most widely recognized biomarkers of oxidative DNA damage [12, 13]. This metabolite, mirroring a ROS-damaged guanine mutagenic effect (due to its tendency to preferentially pair with adenine over cytosine during DNA replication, leading to G-to-T point mutation [14]), has been measured in several pathological conditions [15–18]. In particular, studies in patients with atherosclerotic cardiovascular disease highlight that 8-oxodG is significantly associated with both coronary artery disease (CAD) and other types of atherosclerotic diseases (stroke, peripheral artery disease, and carotid atherosclerosis) [19].

Little is known concerning 8-oxodG and cardiac surgery. Here, we report 8-oxodG levels in CAD patients undergoing CABG procedure, before and at different times after surgery, and in control subjects with cardiovascular risk factors and without overt cardiovascular disease (CTR).

As stated by the European Standards Committee on Urinary (DNA) Lesion Analysis (ESCULA), a fully recognized method to determine 8-oxodG is not defined so far [20]. Therefore, to measure this analyte, a simple and reliable liquid chromatography-tandem mass spectrometry (LC-MS/MS) method has been developed and validated and will be presented here. Finally, we report data on the association between urinary levels of 8-oxodG and 8-iso-prostaglandin F_2*α*_ (8-isoPGF_2*α*_), a well-recognized index of lipid peroxidation [21].

## 2. Materials and Methods

### 2.1. Chemicals and Reagents

8-oxodG was purchased from Sigma-Aldrich (St. Louis, MO, USA); ^15^N_5_-8-oxodG was from Cambridge Isotope Laboratories, Inc. (Andover, MA, USA), and 8-iso-PGF_2*α*_ and 8-iso-PGF_2*α*_-d_4_ were from Cayman Chemicals Co. (Ann Arbor, MI, USA). Amicon® Ultra centrifugal filters (Ultracel®-30 K) were purchased from Merck Millipore Ltd. (Cork, Ireland), and Sep-Pak® C18 solid-phase extraction (SPE) cartridges (3 cc, 500 mg) were purchased from Waters (Milford, MA, USA). Purified water was obtained from Milli-Q® Integral system (Merck Millipore Ltd., Cork, Ireland). All other chromatography-grade chemicals were obtained from Sigma-Aldrich.

### 2.2. Study Population

Over a six-month period (January 2011–June 2011), 52 consecutive CAD patients undergoing CABG surgery on extracorporeal circulation (ECC) were enrolled in the present study at Centro Cardiologico Monzino (CCM). Inclusion criteria were as follows: the need of an elective, isolated surgical procedure; age between 18 and 80 years; heart ejection fraction > 30%; normal sinus rhythm; no history of atrial fibrillation; coronary angiography carried out at least five days before the surgical procedure. Exclusion criteria were as follows: renal or liver disease; intake of antioxidant products (e.g., vitamins). Forty subjects with cardiovascular risk factors (e.g., diabetes, obesity, hypertension, smoking habit, and dyslipidemia without overt cardiovascular disease, on the basis of laboratory tests and clinical examination) acted as CTR group.

Informed consent to participate to this observational study, which was approved by CCM Institutional Review Board, was obtained by all subjects. The study protocol conforms to the ethical guidelines of the 1975 Declaration of Helsinki as reflected in a priori approval by the Institution's Human Research Committee.

### 2.3. Study Protocol and Sample Collection and Preparation

CTR's urinary samples were collected at the scheduled visit. To assess the effect of CABG intervention on oxidative-induced DNA damage in CAD patients, we collected urine samples before (T0), five days (T1), and six months after (T2) CABG procedure. Both in CTR and patients, all urine specimens for 8-oxodG measurement were collected early in the morning, aliquoted in tubes, and stored at −80°C until analysis. Frozen urine were thawed at room temperature, heated at 37°C for 10 minutes to redissolve possible analyte precipitates [22, 23], and then centrifuged at 1,700 ×g for 10 minutes. Subsequently, 200 *μ*L aliquots were diluted with 200 *μ*L of ^15^N_5_-8-oxodG internal standard solution (final concentration 5 ng/mL) and filtered through a 30,000 NMWL (nominal molecular weight limit) centrifugal filters at 10,000 ×g for 30 minutes. The filtrate was injected into the LC–MS/MS system.

For 8-iso-PGF_2*α*_, the processing of the collected urine was previously published [24].

### 2.4. 8-oxodG Measurement

An LC–MS/MS method was set up and validated to measure urinary 8-oxodG levels. The analytical instrument was an Accela HPLC System (Thermo Fisher Scientific, San Jose, CA, USA) coupled to a triple-quadrupole mass spectrometer TSQ Quantum Access (Thermo Fisher Scientific) outfitted with electrospray ionization (ESI) source operating in positive mode. The chromatographic separation was performed using a pentafluorophenyl Kinetex F5 100 Å analytical column (100 × 2.1 mm, Phenomenex, Torrance, CA, USA) packed with 2.6 *μ*m core-shell particles, maintained at 30°C. The mobile phase was set at a flow rate of 0.25 mL/min using ammonium acetate 10 mmol/L (solvent A) and ammonium acetate 10 mmol/L in acetonitrile/water 50 : 50 *v*/*v* (solvent B). Samples (10 *μ*L) were eluted with a gradient of mobile phase (Supplementary Table 1 available online at https://doi.org/10.1155/2017/9715898) during a total run time of 14 minutes. The selected reaction monitoring (SRM) was performed by monitoring the transitions *m*/*z* 284.0 ➔ *m*/*z* 168.1 (8-oxodG) and *m*/*z* 289.0 ➔ *m*/*z* 173.0 (^15^N_5_-8-oxodG). The operating conditions for MS analysis were the following: spray voltage, 2200 V; capillary temperature, 280°C; sheath gas, 25 UA; auxiliary gas, 10 UA. The Xcalibur® software, version 2.0 (Thermo Fisher Scientific), was used for system control, data acquisition, and processing. The method validation was based on U.S. Food and Drug Administration [25] and Matuszewski et al. [26] guidelines, including the evaluation of imprecision, linearity range, lower limit of quantification (LLOQ), limit of detection (LOD), relative matrix effect (ME), extraction recovery (RE), process efficiency (PE), and sample stability. Validation process details are reported in Supplementary Material.

### 2.5. 8-iso-PGF_2*α*_ Measurement

8-Iso-PGF_2*α*_ determination was performed by modifying a previously described LC-MS/MS method [24]. Briefly, the chromatographic separation was performed using an XBridge® C18 column (100 × 2.1 mm, particle size 3.5 *μ*m; Waters Milford, MA, USA) maintained at 30°C. The mobile phase was composed by two solvents: water with 0.1% ammonium hydroxide (solvent A) and methanol : acetonitrile 50 : 50 *v*/*v* with 0.1% ammonium hydroxide (solvent B). The following gradient was used: 0 min—15% B, 14 min—50% B, 16 min—90% B, 22 min—15% B, and 40 min—15% B. SRM was performed in negative mode by monitoring the transitions *m*/*z* 353.1 ➔ *m*/*z* 192.8 (8-iso-PGF_2*α*_) and *m*/*z* 357.05 ➔ *m*/*z* 197.1 (8-iso-PGF_2*α*_-d_4_).

The estimated analyte values were corrected for the urinary creatinine levels, to control the variation in urinary output, and expressed as ng/mg of creatinine (8-oxodG) or pg/mg of creatinine (8-iso-PGF_2*α*_) [27]. Urinary creatinine was measured by a standard method in the clinical laboratory of CCM using Jaffe's reaction.

### 2.6. Statistical Analysis

Continuous variables are presented as mean ± standard deviation (SD) and were compared using the *t*-test for independent samples. Categorical variables were compared using chi-square test or Fisher's exact test, as appropriate.

As 8-oxodG and 8-iso-PGF_2*α*_ values showed a nonnormal distribution, they were log transformed and their time courses were evaluated by covariance analysis for repeated measures. Data were adjusted with multivariable models for age and sex and expressed as geometric mean (GM) with 95% confidence intervals (95% CI). The Pearson correlation was used to detect correlations between 8-oxodG and 8-iso-PGF_2*α*_ at T0. A *p* value <0.05 was considered statistically significant.

All analyses were performed using SAS version 9.4 (SAS Institute, Cary, North Carolina).

## 3. Results

### 3.1. Population

Demographic, clinical, and laboratory features of the two study groups are reported in Table 1. CAD patients were more hypercholesterolemic and hypertensive than CTR subjects; however, total cholesterol, LDL, and HDL were lower in the patient group as a result of the ongoing pharmacological treatments. There was no correlation between 8-oxodG levels and clinical parameters of the studied population.

### 3.2. 8-oxodG Method Validation

**Figure 1:**
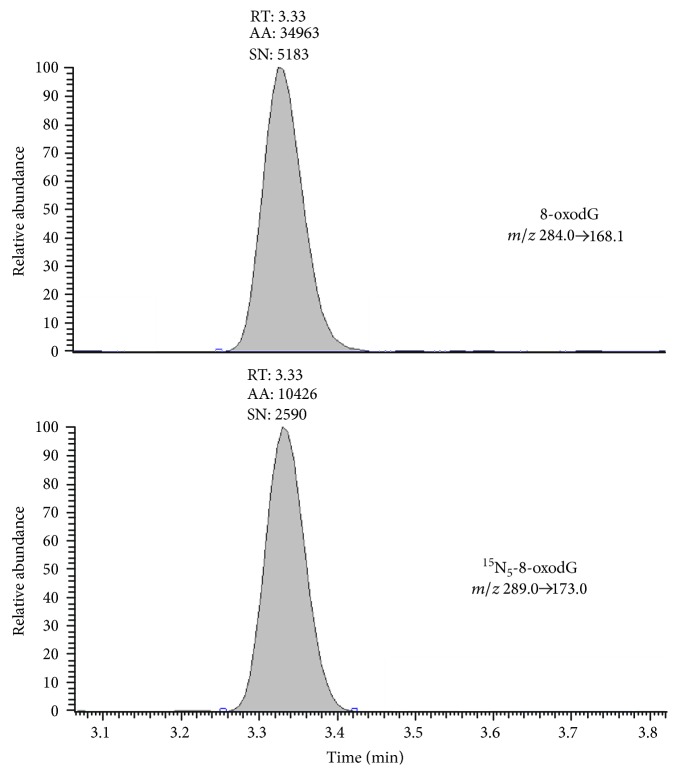
Representative chromatogram of 8-oxodG and of its internal standard ^15^N_5_-8-oxodG in urine pool sample.

A representative chromatogram of 8-oxodG and of its internal standard ^15^N_5_-8-oxodG in urine pool sample, resulting from the chromatographic conditions and the selected transitions, is shown in Figure 1. The peaks eluted at 3.33 min, in a region of the chromatogram without any interfering background peaks.

The 10-point calibrator concentrations, plotted against the ratio of analyte/internal standard areas for five consecutive assays, showed linear and reproducible curves with the following nonzero forced linear regression equations: *y* = (0.1609 ± 0.006) *x* − (0.0143 ± 0.035) (*r*^2^ = 0.999). Over the entire concentration range of the curve, the mean-observed percentage deviation of back-calculated concentrations was between −0.7% and +3.8% with an imprecision coefficient of variation (CV) < 15%. However, as the human urine 8-oxodG concentration seldom exceeds 20 ng/mL, samples quantification was performed using a calibration curve in the range 0.25–25 ng/mL.

Intra-assay and interassay imprecisions were <10% for all quality controls (QC) tested; the LLOQ was 0.25 ng/mL while LOD value was 0.1 ng/mL. Detailed information are provided in Supplementary Table 2.

Supplementary Table 3 shows the relative ME, the RE, and the overall PE of the method. No relative matrix effect was observed at the three concentrations evaluated. Recovery and process efficiency values complied with the acceptability requirements, indicating a good reliability of the developed method.

The analyte was highly stable in urine at different temperatures of storage for at least 6 months and even throughout three freeze-thaw cycles (Supplementary Table 4).

### 3.3. 8-oxodG Levels in CAD Patients Undergoing CABG Surgery

**Figure 2:**
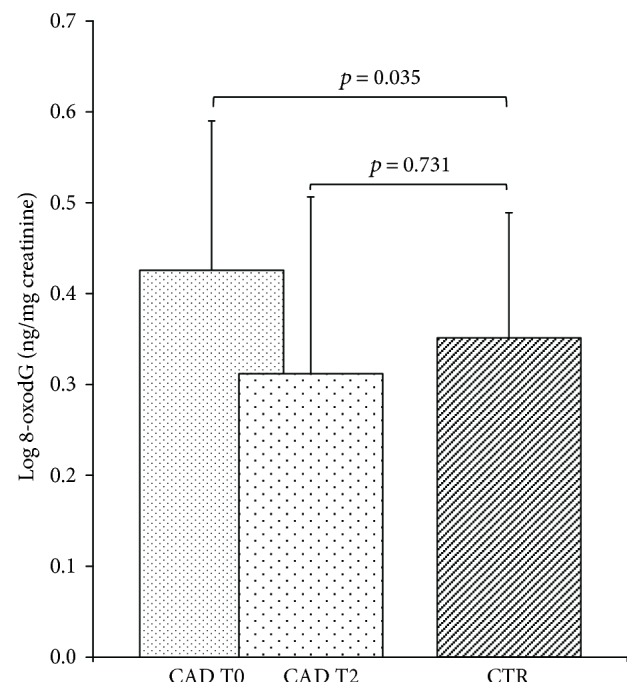
Levels of 8-oxodG measured in urine from CAD patients and CTR subjects. Data are represented as mean ± SD. Comparisons between groups were performed by covariance analysis, adjusting for age and sex.

In Figure 2 the 8-oxodG levels in CAD and CTR are depicted. As shown, the values of 8-oxodG measured in CAD patients before surgery (T0) (GM: 2.66 ng/mg creatinine, 95% CI: 2.39–2.95), were significantly higher than those found in CTR (GM: 2.26 ng/mg creatinine, 95% CI: 2.00–2.56) (*p* = 0.035). In contrast, six months after the intervention (T2) (Figure 2), 8-oxodG levels of CAD patients were entirely comparable to those of CTR (GM: 2.04 ng/mg creatinine, 95% CI: 1.84–2.27 and 2.26 ng/mg creatinine, 95% CI: 2.00–2.56, resp.; *p* = 0.73).

**Figure 3:**
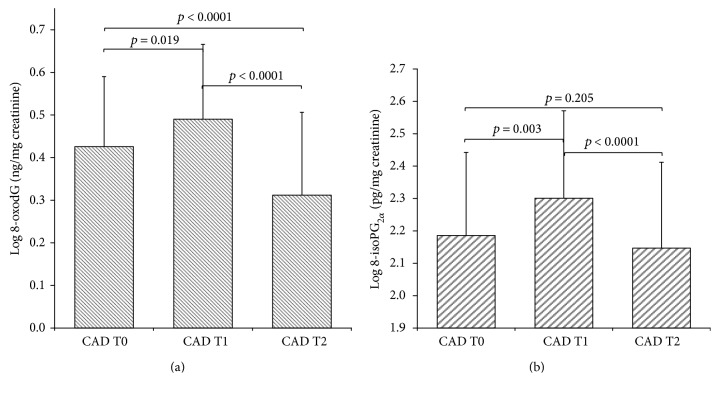
Time course of 8-oxodG (a) and of 8-isoPGF_2*α*_ (b) following CABG surgery. Data are represented as mean ± SD.

8-oxodG values at the different time points are shown in Figure 3(a). There was a statistical significant increase of short-term oxidative DNA damage after cardiac surgery (delta T1-T0, *p* = 0.019) and a significant decrease after six months from the intervention (delta T2-T1, *p* < 0.0001) with a full recovery at T2 (delta T2–T0, *p* < 0.0001), up to normal values.

No correlation between 8-oxodG levels and ECC or clamping time (*r* = 0.115, *p* = 0.42, and *r* = 0.205, *p* = 0.1, resp.) was observed.

### 3.4. 8-Iso-PGF_2*α*_ Levels in CAD Patients Undergoing CABG Surgery

Levels of 8-iso-PGF_2*α*_ in CAD patients undergoing surgery are depicted in Figure 3(b). Similar to 8-oxodG, an increase in 8-iso-PGF_2*α*_ values was observed following the surgical procedure (delta T1-T0, *p* = 0.003) that returned to basal levels six months later (delta T2-T1, *p* < 0.0001). However, no significant difference was found before and six months after CABG procedure (delta T2–T0, *p* = 0.205).

At variance with 8-oxodG, no significant differences were found in 8-iso-PGF_2*α*_ values between CTR (GM: 141.2 pg/mg creatinine 95% CI: 117.25–170.06) and CAD patients before surgery (T0) (GM: 155.06 pg/mg creatinine 95% CI: 132.45–181.53).

### 3.5. Correlation between 8-iso-PGF_2*α*_ and 8-oxodG Levels

**Figure 4:**
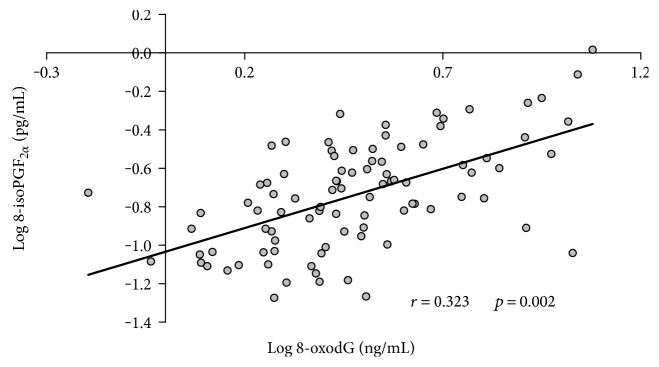
Correlation between baseline levels of 8-oxodG and 8-iso-PGF_2*α*_ in the whole population analyzed (*n* = 92).

As shown in Figure 4, at baseline, a direct and positive correlation was found in the whole population studied (*n* = 92) between the two oxidative stress markers considered, 8-oxodG and 8-iso-PGF_2*α*_ (*r* = 0.323, *p* = 0.002).

## 4. Discussion

The aim of this study was to assess the effect of CABG procedure on the oxidative stress status in CAD patients. To reach this goal, we decided to measure 8-oxodG, a marker of oxidative DNA damage, 5 days and 6 months after CABG surgery. For this purpose, we developed an LC-MS/MS method to quantify urinary 8-oxodG level. Although LC-MS/MS is the gold standard technique to measure 8-oxodG [27–29], and various methods are currently employed (as stated by ESCULA), none of them is, up to now, overall accepted [20]. The LC-MS/MS method that we have developed and validated is simple, sensitive, and reliable and can be proposed in routine determination of 8-oxodG levels.

Following the application of this method, we show that, before CABG procedure, CAD patients have significantly higher urinary 8-oxodG levels as compared to CTR. Previously published studies in different matrices (serum [30], DNA extracts from leukocyte or lymphocytes [31, 32], and urine [33, 34]) showed higher than normal 8-oxodG concentrations in atherosclerotic patients. Moreover, a previously published meta-analysis [19] documented a statistically significant higher concentrations of 8-oxodG in CAD patients than in control subjects. In keeping with this, enhanced 8-oxodG concentrations have been reported in atherosclerotic plaques compared to the underlying media or to nonatherosclerotic mammary arteries [35] and to elevated DNA strand breaks in cells isolated from the atherosclerotic lesions [36]. 8-oxodG measurement in cardiovascular disease allows to evaluate oxidative damage from a different point of view, catching a picture of ROS-induced mutagenic effect. Indeed, as previously discussed, 8-oxodG reflects mutagenic guanine damage that may mispair with adenine, leading to a G-to-T point mutation during replication [14].

The return of 8-oxodG concentrations toward control values in CAD patients six months after cardiac surgery reveals the recovery of oxidative stress toward physiological levels. It is conceivable that CABG procedures, probably in association with pharmacological therapy, reduce oxidative stress and, in turn, ischemia-related DNA damage in CAD patients. Established atherogenic risk factors such as hypertension, smoking habits, and diabetes mellitus have been associated to an increased oxidative stress [37]; however, the pre- and postsurgical schedules for the treatment of these risk factors did not change in the present setting.

The present data also provide evidence for a short-term burst of 8-oxodG associated with CABG surgery, highlighting a significant increase of this metabolite 5 days after procedure. A higher than normal oxidative stress has been associated to CABG, and it is considered a risk factor for postoperative complications such as morbidity and mortality, in this very frequent cardiac intervention [1]. The observed increase in oxidative DNA damage and oxidative stress at T1 is likely to reflect the overproduction of ROS due to surgical trauma and/or ischemia-reperfusion process. The contact of blood with polymers of the extracorporeal circuit may also induce neutrophil activation and, in turn, ROS production [38]. However, we did not observe any correlation between ECC or clamping time and 8-oxodG levels in the present setting. Karahalil et al. assessed the short-term effects of CABG surgery on 8-oxodG serum levels [9], but they did not observe any difference in the levels of 8-oxodG in samples obtained before versus after surgery. Differences in the matrices and in the collection times employed may well explain the discrepancies between the present data and those obtained by Karahalil et al.

Our results clearly highlight that CABG surgery causes a burst of oxidative damage both in DNA and lipids (as depicted by the increase of 8-oxodG and 8-isoPGF_2*α*_, resp.). In line with other studies in this area [39–41], we have found a direct positive baseline correlation between urinary 8-isoPGF_2*α*_ and 8-oxodG in samples obtained from CAD patients and CTR. However, England and coworkers showed no correlation between these two markers in healthy subjects [42]. These authors measured these metabolites in white cells (as to 8-oxodG) and in plasma (as to 8-isoPGF_2*α*_), leading to the hypothesis that measuring both analytes in the same matrix may probably give a more reliable assessment of the in vivo oxidative stress status and an explanation for the discrepancy between the two data.

In addition to similarities, these two markers of oxidative stress also show potential differences in the oxidative stress evaluation. The fact that, at variance with 8-oxodG, 8-iso-PGF_2*α*_ levels were entirely comparable to those obtained 6 months after surgery deserves some considerations. Being an index of lipid peroxidation, 8-iso-PGF_2*α*_ levels are likely to be influenced by plasma cholesterol levels and by the ongoing treatments on total and LDL cholesterol [43]. Table 1 indicates that plasma total, LDL, and HDL cholesterol are significantly higher in CTR than in CAD patients, a finding consistent with an ongoing, probably long-term, statin treatment (as also reported in Table 1) and a more intensive lipid-lowering strategy of (pharmacological/nutritional) intervention in the present CAD setting. In addition, hypertension [44], smoking habits, [45] and diabetes [46] (recognized cardiovascular risk factors) show a positive correlation with 8-iso-PGF_2*α*_ and their presence in the CTR could mask the differences between the groups.

Our study has some potential limitations. The controls employed do not entirely match the clinical characteristics of the 52 subjects that underwent CABG surgery. However, consecutive patients were chosen, and the inclusion/exclusion criteria employed were stringent enough to avoid major biases. Another possible limitation is the small number of subjects enrolled. Future investigation in the area, with larger numbers of patients, will help to confirm and extend the clinical impact of the present findings.

## 5. Conclusion

The LC-MS/MS method developed and validated in our laboratory allows monitoring the oxidative DNA damage without interfering with patient's postoperative progress, being urine as one of the simplest biological matrices. The results obtained in this study highlight a short-term oxidative DNA damage induced by CABG procedure in CAD patients and a complete recovery of this oxidative burst six months after the procedure.

Overall, our results argue for normalization of oxidative DNA damage vis-à-vis correction of ischemia-related ROS damage in CAD patients.

## Supplementary Material

8-oxodG method validation procedures. Supplemental Table 1. Mobile phase gradient. Supplemental Table 2. Imprecision, LLOQ and LOD of the LC-MS/MS method. Supplemental Table 3. Matrix effect, recovery and process efficiency data for 8-oxodG in urine. Supplemental Table 4. Stability of 8-oxodG in urine.

## Figures and Tables

**Table 1:** Overall population characteristics at baseline.

Variables	CAD (*n* = 52)	CTR (*n* = 40)	*p* value
*Demographic characteristics*			
Age, years	63.5 ± 9.7	58.5 ± 7.0	0.004
Male gender, number (%)	40 (76.9)	22 (55.0)	0.014
*Clinical characteristics*			
Weight, kg	75.6 ± 13.8	78.1 ± 13.7	0.390
BMI, kg/m^2^	26 ± 5.2	27.5 ± 4.1	0.080
Active smokers, number (%)	6 (11.5)	6 (15)	0.630
*Biochemical*			
Total cholesterol, mg/dL	190.7 ± 44.2	212.5 ± 29.0	0.004
LDL cholesterol, mg/dL	121.8 ± 35.6	139.5 ± 32.4	0.020
HDL cholesterol, mg/dL	48.0 ± 10.6	52.7 ± 12.9	0.004
Triglycerides, mg/dL	104.0 ± 41.6	130.3 ± 82.4	0.070
Fasting glycemia, mg/dL	117.7 ± 28.1	107.0 ± 31.3	0.190
Creatinine, mg/dL	149.9 ± 77.3	125.2 ± 51.8	0.070
*Comorbidities*			
Diabetes, number (%)	7 (13.5)	1 (2.5)	0.080
Hypertension, number (%)	48 (92.3)	17 (42.5)	0.005
Hypercholesterolemia, number (%)	29 (55.8)	9 (22.5)	0.001
Obesity, number (%)	1 (1.9)	5 (12.5)	0.070
*Medications*			
Aspirin, number (%)	33 (63.5)	0 (0)	*p* < 0.001
Statins, number (%)	28 (53.9)	4 (10)	*p* < 0.001
Oral hypoglycemics, number (%)	7 (13.5)	1 (2.5)	0.160
Antihypertensive, number (%)	48 (92.3)	13 (32.5)	*p* < 0.001

Characteristics of the study groups: CAD patients (*n* = 52) and CTR subjects (*n* = 40). Values are presented as mean ± SD or in absolute numbers (percentage of total).

## Abbreviations

8-isoPGF_2*α*_: 8-Iso-prostaglandin F_2*α*_
8-oxodG: 8-Oxo-7,8-dihydro-2′-deoxyguanosine
CABG: Coronary artery bypass grafting
CAD: Coronary artery disease
CCM: Centro Cardiologico Monzino
CI: Confidence interval
CTR: Control subject
ECC: Extracorporeal circulation
GM: Geometric mean
HDL: High-density lipoprotein
LC–MS/MS: Liquid chromatography-tandem mass spectrometry
LDL: Low-density lipoprotein
LLOQ: Lower limit of quantification
LOD: Limit of detection
ME: Matrix effect
PE: Process efficiency
QC: Quality control
RE: Extraction recovery
SD: Standard deviation
SRM: Selected reaction monitoring.
