# Supplementary material for: Assessing Free-Radical-Mediated DNA Damage during Cardiac Surgery: 8-Oxo-7,8-dihydro-2′-deoxyguanosine as a Putative Biomarker
Source: Oxid Med Cell Longev. 2017 Jun 4;2017:9715898. doi: 10.1155/2017/9715898 (PMC5474244; doi:10.1155/2017/9715898)
Supplement: Supplementary file 1 — 8-oxodG method validation procedures. Supplemental Table 1. Mobile phase gradient. Supplemental Table 2. Imprecision, LLOQ and LOD of the LC-MS/MS method. Supplemental Table 3. Matrix effect, recovery and process efficiency data for 8-oxodG in urine. Supplemental Table 4. Stability of 8-oxodG in urine. [file 9715898.f1.docx]

**SUPPLEMENTAL MATERIAL**

**Assessing free-radical-mediated DNA damage during cardiac surgery:**

**8-oxo-7,8-dihydro-2’-deoxyguanosine as a putative biomarker**

Linda Turnu*^1^, Alessandro Di Minno*^1^, Benedetta Porro^1^, Isabella Squellerio^1^, Alice Bonomi^1^, Chiara Maria Manega^1^, José Pablo Werba^1^, Alessandro Parolari^2^, Elena Tremoli^1^, Viviana Cavalca^1^

*The two Authors equally contributed to the present report

^1^ Centro Cardiologico Monzino, IRCCS, Milan, Italy;

^2^ Dipartimento di Chirurgia cardiaca, IRCCS Policlinico San Donato, Milan, Italy

| **TITLE** | **PAGE** |
| --- | --- |
| **8-oxodG method validation procedures** | **2** |
| **Supplemental Table 1. Mobile phase gradient** | **4** |
| **Supplemental Table 2. Imprecision, LLOQ and LOD of the LC-MS/MS method** | **4** |
| **Supplemental Table 3. Matrix effect, recovery and process efficiency data for 8-oxodG in urine** | **4** |
| **Supplemental Table 4. Stability of 8-oxodG in urine** | **5** |
| **References** | **6** |
|  |  |

**8-oxodG method validation procedures**

Ten human urine samples were pooled together (pooled urine, **PU**), aliquoted and used in the assay performance evaluation following the U.S. Food and Drug Administration guidelines[[1](#_ENREF_1)].

Due to the lack of a “blank” urine sample, the assessment of assay imprecision was checked with quality controls (**QC**) prepared using PU: low concentration QC was made by diluting PU 1:2 v/v with water, medium and high QC were prepared by fortifying the endogenous 8-oxodG PU concentration with known amounts of the standard solution (spiked concentrations at 2 ng/mL and 25 ng/mL for medium and high QC respectively). The intra-assay imprecision was determined by assaying 9 separate aliquots of each QC sample in a single batch. Inter-assay imprecision was determined by testing a single aliquot of each QC in 5 consecutive days.

The linearity and range of the calibration curve were evaluated with ten standard calibrators over the concentration range 0.1-100 ng/mL. Each calibrator was spiked with internal standard ^15^N_5_-8-oxodG (final concentration 5 ng/mL). Linearity of the assay was assessed by repeated analysis (n=5) of calibrators and linear regression analysis was used to determine the slope, intercept, and correlation coefficient (r^2^). The lower limit of quantification (**LLOQ)** was calculated as the lowest concentration providing a coefficient of variation (**CV**) <20% and an accuracy between 80% and 120%. The limit of detection (**LOD**) was defined as the lowest concentration that gave a signal-to-noise of at least 3.

The stability of the analyte at different temperatures was tested by analysing PU aliquots kept at -80°C; -20°C, +4°C and +21°C for 24h, 72h and 6 months.

For the freeze/thaw stability study, two aliquots of PU were used. The first aliquot was immediately analysed for the quantification while the second was frozen at -80°C and assayed after being freeze/thawed three times in three consecutive days. The stability is defined as percentage related to sample analyzed immediately after collection.

Relative matrix effect (**ME**), extraction recovery (**RE**), and process efficiency (**PE**) were evaluated according to Matuszewski et al[[2](#_ENREF_2)]. All these parameters were assessed at three different concentrations of 8-oxodG (0.5, 5 and 50 ng/mL added) and the analysis was repeated 5 different times. Three sets of each concentration levels were prepared as follows: neat 8-oxodG standard solution (**set A**); PU samples spiked with 8-oxodG standard after centrifugal filtration (**set B**); PU samples spiked with 8-oxodG standard before centrifugal filtration (**set C**). The values of set A, B and C are expressed, in arbitrary units, as mean of area ratio. For set B and C, the spiked area ratio of 8-oxodG was calculated by subtracting the basal endogenous 8-oxodG value (unspiked) from the measured area ratio. The rates of ME, RE and the overall PE were determined at each concentration tested as follows: ME (%) = peak area ratio from set B/peak area ratio from set A × 100; RE (%) = peak area ratio from set C/peak area ratio from set B × 100; PE (%) = peak area ratio from set C/peak area ratio from set A × 100.

**Supplemental Table 1. Mobile phase gradient**

**Supplemental Table 2. Imprecision, LLOQ and LOD of the LC-MS/MS method**

**Supplemental Table 3. Matrix effect, recovery and process efficiency data**

**for 8-oxodG in urine**

**Supplemental Table 4. Stability of 8-oxodG in urine**

**References**

1. U.D.o.H.a.H.S. Food and Drug Administration F, Center for Drug Evaluation and Research. Guidance for Industry: Bioanalytical Method Validation, <http://www.fda.gov/downloads/drugs/guidancecomplianceregulatoryinformation/guidances/ucm368107.pdf2013>.

2. Matuszewski BK, Constanzer ML, Chavez-Eng CM. Strategies for the assessment of matrix effect in quantitative bioanalytical methods based on HPLC-MS/MS. Analytical chemistry. 2003;75(13):3019-30. Epub 2003/09/11.
